# Supplementary material for: Integrated Analysis of Transcriptome and Metabolome Provides Insight into Camellia oleifera Oil Alleviating Fat Accumulation in High-Fat Caenorhabditis elegans
Source: Int J Mol Sci. 2023 Jul 19;24(14):11615. doi: 10.3390/ijms241411615 (PMC10380387; doi:10.3390/ijms241411615)
Supplement: Supplementary file 1 [file ijms-24-11615-s001.zip › Figure S1.pdf]

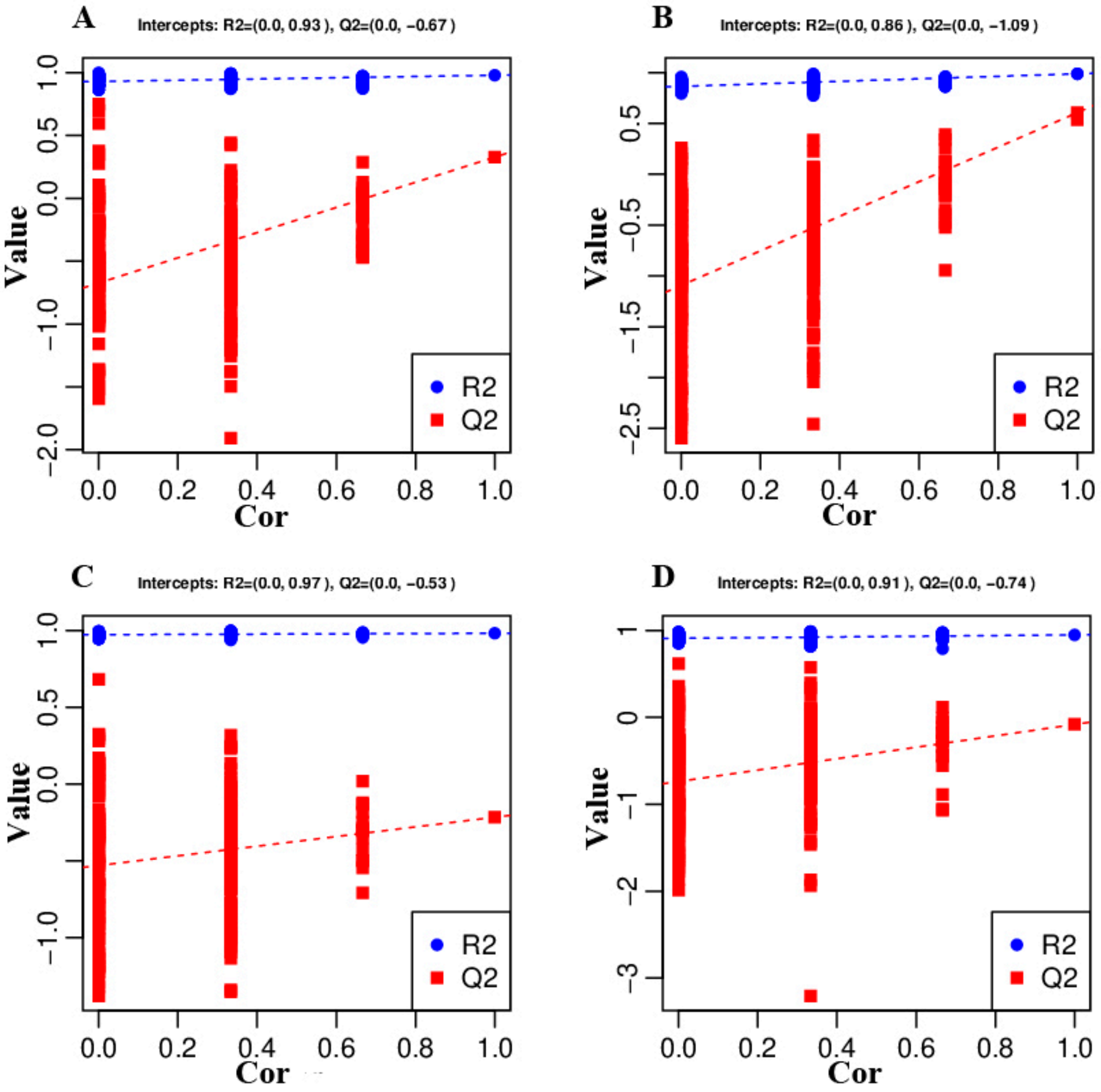

Figure S1. The corresponding R^2 and Q^2 values of PLS-DA. (A) Glc/Control pos, (B) Glc/Control neg, (C) Glc-CO/Glc pos, (D) Glc-CO/Glc neg.
